# P-1663. The effectiveness of forward contact tracing and backward source investigation in controlling COVID-19 transmission: a case-series study in Yamagata, Japan, between 2020–2022

**DOI:** 10.1093/ofid/ofaf695.1838

**Published:** 2026-01-11

**Authors:** Takeaki Imamura, Yoshiharu Mori, Shunji Fujii, Emiko Suzuki, Keiko Yamada, Yoshihiro Ashino, Yuichi Kato, Hidetoshi Yamashita, Junji Seto, Mayuko Saito, Taro Kamigaki, Tomoe Shimada, Motoi Suzuki, Katsumi Mizuta, Tadayuki Ahiko, Hitoshi Oshitani

**Affiliations:** Tohoku University, Sendai, Miyagi, Japan; Murayama Public Health Center, Yamagata, Yamagata, Japan; Murayama Public Health Center, Yamagata, Yamagata, Japan; Mogami Public Health Center, Yamagata, Yamagata, Japan; Okitama Public Health Center, Yamagata, Yamagata, Japan; Shonai Public Health Center, Yamagata, Yamagata, Japan; Yamagata City Institute of Public Health, Yamagata, Yamagata, Japan; Yamagata City Institute of Public Health, Yamagata, Yamagata, Japan; Yamagata Prefectural Institute of Public Health, Yamagata, Yamagata, Japan; Department of Virology, Graduate School of Medicine, Tohoku University, Sendai, Miyagi, Japan; Japan Institute for Health Security, Shinjuku, Tokyo, Japan; National Institute of Infectious Deaseases, chiyoda-ku, Tokyo, Japan; Japan Institute for Health Security, Shinjuku, Tokyo, Japan; Yamagata Prefectural Institute of Public Health, Yamagata, Yamagata, Japan; Health and Welfare Department, Yamagata Prefectural Government, Yamagata, Yamagata, Japan; Department of Global Health and Infectious Diseases, Graduate School of Medicine, Tohoku University, Sendai, Miyagi, Japan

## Abstract

**Background:**

The real-world data regarding the effectiveness of forward contact tracing and backward source investigation in controlling COVID-19 transmission remains limited.

**Methods:**

We reviewed records of forward contact tracing and backward source investigation conducted in four public health centers (PHCs) in Yamagata, Japan, regarding COVID-19 cases reported between January 2020 and February 2022. A transmission setting was defined as where an epidemiological link occurred, and onward transmission as a transmission from one transmission setting to another. Cases identified and requested to stay at home based on PHCs’ contact tracing and source investigation were classified into the Intervention group, cases who took COVID-19 tests and stayed at home upon diagnosis without PHCs’ intervention into the Non-intervention group, and others into the Partial-intervention group. This study was approved by Yamagata Prefectural Institute of Public Health (No. 125), Tohoku University (2023-1-252), and the National Institute of Infectious Diseases (No. 1595), Japan.

**Results:**

Among 8,627 COVID-19 cases reported in four Yamagata PHCs, transmission settings were identified in 6,517 cases (76%). Of 6,517 cases, 3,947 cases (61%) were classified into the Intervention, 2,199 cases (34%) into the Non-intervention, and 371 cases (6%) into the Partial-intervention group. The interval between symptom onset and the stay-at-home requests was 0 days [IQR: -1 – 2] among the Intervention group, which was shorter compared to that among the Non-intervention (2 days [IQR: 1–3], *p< 0.001*) and the Partial-intervention group (1 day [IQR: 0–2], *p< 0.001*). Cases belonging to the Intervention (aOR 0.62 [95%CI: 0.52–0.75], *p< 0.001*) and the Partial-intervention group (aOR 0.62 [95%CI: 0.44–0.88], *p=0.007*) were less likely to generate onward transmission compared to those in the Non-intervention group.

**Conclusion:**

In this study, COVID-19 cases with identified transmission settings receiving intervention based on PHCs’ forward contact tracing and backward source investigation were associated with earlier stay-at-home requests and less likelihood of generating onward transmission than cases without contact-tracing/source-investigation-based intervention.

**Disclosures:**

Hitoshi Oshitani, MD, PhD, MPH, Global Coalition On Aging: Steering Committee Member without remuneration|SHIONOGI INFECTIOUS DISEASE RESEARCH PROMOTION FOUNDATION: Board Member

